# Emergence of Exploratory, Technical and Tactical Behavior in Small-Sided Soccer Games when Manipulating the Number of Teammates and Opponents

**DOI:** 10.1371/journal.pone.0168866

**Published:** 2016-12-22

**Authors:** Carlota Torrents, Angel Ric, Robert Hristovski, Lorena Torres-Ronda, Emili Vicente, Jaime Sampaio

**Affiliations:** 1 National Institute of Physical Education of Catalonia (INEFC), University of Lleida, Lleida, Spain; 2 Sts. Cyril and Methodius University, Skopje, Macedonia; 3 Department of Health and Kinesiology, Exercise and Sport Nutrition Lab, Texas A&M University, College Station, Texas, United States of America; 4 CreativeLab, Research Center in Sports Sciences, Health Sciences and Human Development (CIDESD), Vila Real, Portugal; 5 Sport Sciences Department, Universidade de Trás-Os-Montes e Alto Douro, Vila Real, Portugal; University of Lethbridge, CANADA

## Abstract

The effects that different constraints have on the exploratory behavior, measured by the variety and quantity of different responses within a game situation, is of the utmost importance for successful performance in team sports. The aim of this study was to determine how the number of teammates and opponents affects the exploratory behavior of both professional and amateur players in small-sided soccer games. Twenty-two professional (age 25.6 ± 4.9 years) and 22 amateur (age 23.1 ± 0.7 years) male soccer players played three small-sided game formats (4 vs. 3, 4 vs. 5, and 4 vs. 7). These trials were video-recorded and a systematic observation instrument was used to notate the actions, which were subsequently analyzed by means of a principal component analysis and the dynamic overlap order parameter (measure to identify the rate and breadth of exploratory behavior on different time scales). Results revealed that a higher the number of opponents required for more frequent ball controls. Moreover, with a higher number of teammates, there were more defensive actions focused on protecting the goal, with more players balancing. In relation to attack, an increase in the number of opponents produced a decrease in passing, driving and controlling actions, while an increase in the number of teammates led to more time being spent in attacking situations. A numerical advantage led to less exploratory behavior, an effect that was especially clear when playing within a team of seven players against four opponents. All teams showed strong effects of the number of teammates on the exploratory behavior when comparing 5 vs 7 or 3 vs 7 teammates. These results seem to be independent of the players’ level.

## Introduction

Tactical creativity refers to varying, atypical and flexible decisions and actions, and it plays a key role in team ball sports due to the complex and chaotic determinants of performance [1,2]. Coaches are especially interested in developing a more flexible game to increase unpredictable behavior through emergent properties [3] and gain advantage over the opponents [1]. Tactical creativity can be described by the classical characteristics of creativity defined by Guilford [4]: the unusualness of tactical actions defines originality; the variety of tactical actions defines flexibility; and the number of tactical actions generated for certain situations defines fluency [5]. Therefore, it is important to design practice tasks that can foster players’ creative behavior and, consequently, improve performance outcomes. Despite this, few studies have addressed the topic of creativity in sport performance, and there remains a need to establish solid modeling approaches for studying and explaining creative behavior [6,7].

The most widely used definitions of creativity refer to obtaining a useful and original product or behavior [8–13], however, several authors with closer ties to education have particularly focused the concept on individual and exploratory behavior. This conceptualization of creativity classifies as creative any exploratory behavior without regard to whether it leads to a novel final outcome or not [14]. Exploratory behavior may be defined as a subsequent realization of a large number of movement configurations under specific constraints of each performer [15]. Therefore, exploratory activity is strongly dependent on the set of constraints imposed on the system. This observation implies that during training programs no explicitly prescriptive list of possible actions needs to be provided beforehand to the performer so that s/he might realize them [6]. This approach is relevant to team sports, which are based on improvisation and on the interaction between the performers and the environment. The exploratory behavior that emerges in team sports will depend on the affordances of the individuals involved, and on the influence of the environment. Affordances have been defined as opportunities [16], invitations [17] or solicitations [18] for action, but always as a scaled relation between certain properties of the environment and a performer’s effectiveness, i.e. abilities [19,20]. It is the affordances that channel the exploratory activity of the performer. The interrelatedness of affordances [18] allows us to understand the emergence of behavior at different levels of game constraints. Therefore, it may be necessary to provide task constraints allowing players to adapt constantly their behavior to the unpredictable opponents’ behavior. Practice tasks should seek to promote varied and flexible behavior so that players learn to be more adaptive, and the inclusion of exploratory tasks can help them to be more creative.

A coordination dynamics approach, based on complex systems theories and ecological psychology, offers a useful theoretical explanation of behavior based on performer-environment relationships [21,22]. Under this approach, individuals and teams can be modeled as complex social and nondeterministic systems [23]. From this perspective, functional patterns of coordinated behavior emerge from ongoing interactions between system components through a process of self-organization between players, the environment, and specific task constraints [24].

Hristovski, Davids, and Araújo [25] showed how the manipulation of task constraints might enable the emergence of innovative and functional tactical behaviors. In boxing, it was observed that changing the distance of the participants to a punching bag led to spontaneous and novel behaviors, in this case, specific striking techniques that had not been practiced or taught before. It was also shown how the spontaneous and innovative behaviors emerge only for some specific task constraints. Extremes of very high and very low risk-of-being-hit constraints were suppressing the innovative behavior and only medium cost constraints enabled emergence of novel actions. Torrents, Ric, and Hristovski [26] showed how the manipulation of task constraints in dancers had a significant effect on the type of configurations performed, as well as on their explorative behavior (i.e., the degree of exploration of dancers’ movement/postures). Unusual rules resulted in a greater exploration of movement possibilities. Moreover, results showed that novel dancers explored a significantly smaller region of the potential landscape than experts did, suggesting that this measure may help measuring the dancers’ performances [27]. These observations of the emergence of human innovative behaviors are important from the perspective of Boden’s [28] definition of transformational creativity, since they explain how manipulating constraints, enables a transformation within a neurobiological action system without reference to abstract rule-governed behaviors. Traditionally, coaches design partial and separate practice exercises for technical, physiological, and tactical aspects, and in some cases there is little correspondence with actual game situations [29]. However, coaches need to develop practice scenarios that are representative of the actual competition in order to achieve a better coupling between perception and action during the games [30].

In soccer, a common way to modify task constraints is implementing small-sided games, a training task widely described in the literature [31,32]. These training scenarios exploit inherent self-organizing tendencies and can simultaneously develop technical skills (related with the movement of players’ body with the ball) and tactical behaviors (spatial-temporal movements related with the shared task-goals in offense or defense) in competitive performance environments. The game is unpredictable and helps players maintain higher levels of motivation in comparison with non-specific training situations. During small-sided games without external instructions, the information flows, allowing continuous spontaneous interactions between teammates and opponents [23]. The most common responses analyzed when playing small-sided games have been those related to physiological or physical nature [31,33–35], in addition to technical [36–38] and tactical aspects related to players’ positioning [36, 39]. In particular, considering the number of players involved, Jones and Drust [40], and Katis and Kellis [41] reported that the number of technical actions increased with a decrease in the number of players. However, Abrantes, Nunes, Maçãs, Leite, and Sampaio [42] observed no differences in player effectiveness in relation to the density of players. Similarly, Duarte, Batalha, Folgado, and Sampaio [43] founded that, in semi-professional futsal players, the lower the number of players during the small-sided games, the more frequent the technical actions (e.g., successive contacts with the ball, the number of dribbles, and the number of tackles). Casamichana and Castellano [44] studied the frequency of appearance of technical actions in soccer and found they became more common as the density of players, i.e. individual playing area was reduced. Specifically, there were differences between small (32 x 23 m) and medium-sized (50 x 35 m) pitches for the variables control and shoot, clearance, and putting the ball in play. Between small and large (62 x 44 m) pitches there were also differences for interception, control and dribble, clearance, and putting the ball in play. Owen et al. [45] found differences in the technical-related performance with the ball between small- (4 vs. 4), medium- (5 vs. 5 to 8 vs. 8), and large-sided games (i.e., 9 vs. 9 to 11 vs. 11), where passing, receiving, dribbling, and shots, were more frequent as the number of players decreased. Nevertheless, very little research has studied technical responses in games involving unequal numbers of players, even though this kind of numerical (dis-) advantage is common, and likely to be associated with more game perturbations and, in consequence, more scoring opportunities [32]. Silva et al. [39] evaluated the effect of different numerical relations (5vs5, 5vs4 and 5vs3) on positioning considering inter-individual, intra- and inter-team coordination. They found that these asymmetries constrained players’ individual dominant regions, modified the teams’ dispersion (decreased in inferiority), the teams’ relative positioning on field (approached the opponent’s goal in superiority) and the space between line-forces on wings and sectors.

Although small-sided games are well described in the literature, research related to their effect on the creative behavior of the players is very scarce. In general, very limited research has been conducted so far focused in the development of training methods that may foster creative behavior in sport. Some of the proposals to date include the use of diversification, deliberate play, deliberate practice, or deliberate coaching (see [1] for a review). However, measuring creativity in sport is challenging, and previous research has shown how difficult it can be to reach an agreement about what constitutes an original response [46]. Due to the subjective nature of this aspect of tactical creativity, the present study takes into account criteria related to the variety and quantity of responses (i.e., flexibility and fluency) [4], but does not consider the rarity or originality of responses. For this reason, we will address exploratory behavior instead of tactical creativity to describe the players’ creative behavior. Moreover, we propose unusual training situations with the aim of stimulating greater exploration of the tactical possibilities, considering the suggestion by Stokes [47,48] where in order to produce a creative solution, constraints not only may preclude existing responses, but also promote novel, and frequently, opposites. We hypothesize that playing in difficult scenarios such as playing in numerical inferiority will facilitate the emergence of more varied behavior. We further hypothesize that the modification of the number of opponents and teammates will produce the use of different number and type of technical and tactical actions. Therefore, the aim of this study was to examine how the constraints such as number of opponents and teammates affect the technical, tactical, and exploratory behavior in small-sided games, in both professional and amateur players.

## Materials and Methods

### Participants

Twenty-two professional (PRO) males from a single soccer team (age: 25.6 ± 4.9 years; height: 180.5 ± 4.3 cm; weight: 74.7 ± 4.8 kg), and twenty-two amateur (AMA) male players enrolled in a sports sciences degree (age: 23.1 ± 0.7 years; height: 179.6 ± 6.1 cm; weight: 72.3 ± 5.9 kg) were recruited for this study. Goalkeepers (GK) took part in the games, but were excluded from the analysis. All players were informed about the research procedures, requirements, benefits, and risks, and their written consent was obtained before the study began. The investigation was approved by the local institutional Research Ethics Committee and it conformed to the recommendations of the Declaration of Helsinki.

### Procedure

**Fig 1:**
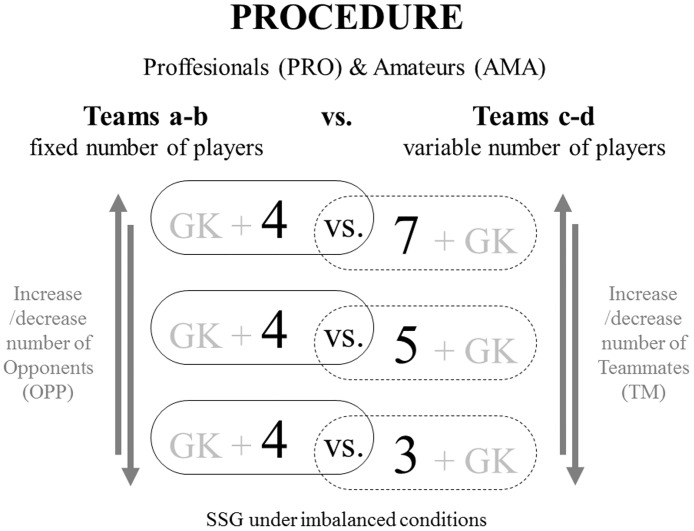
Procedure followed in the data collection.

The study was conducted over two separated sessions, one for PRO and one for AMA, both following the same procedures. The players were distributed into two teams (a, b) with a fixed number of players (4), and two teams (c, d) with a variable number of players (3, 5, or 7). The head coach of the professional team and the teacher of the students was the same person and used subjective evaluation to distribute the players in the different teams. Their physical, technical, and tactical performances were the criteria used to constitute balanced teams [31]. Both sessions involved three different small-sided games: GK + 4 vs. 7 + GK; GK + 4 vs. 5 + GK; and GK+ 4 vs. 3 + GK, lasting 3 min each, with 4 min of passive recovery (Fig 1). These three small-sided games formats were repeated twice, yielding a total of six situations for each team. All these games were played with a numerical mismatch between the teams involved, and in a randomized order. Each team always competed against the same team (i.e., AMAa vs. AMAc; AMAb vs. AMAd; PROa vs. PROc; PROb vs. PROd). Neither professional nor amateur players were familiar with the 4 vs. 7 situations. All the small-sided games were played on a 40 × 30 m artificial turf pitch, and in accordance with the official soccer rules. To encourage high work-rate maintenance, coaches or other players were allowed to give verbal encouragement to all players, but not specific feedback related with players’ performance or their technical/tactical behavior. In addition, several balls were placed around the pitch perimeter with the aim of increasing the effective playing time. In order to avoid the effect of the scoring, the scoreboard turned to 0 when any team got two goals. Each session ended with a 10 min cool-down, consisted of static stretching exercises.

All small-sided games were video-recorded using three cameras, and analyzed by two independent experienced soccer coaches (Graduated in Sports Sciences, certified with UEFA PRO license and with experience as coaches). For all small-sided games, observers took into account the actions and characteristics of the actions performed by both teams. These actions or characteristics were defined on a coarse-grained scale of 51 categories (see Table 1) adapting the proposals of Owen, Twist and Ford [49] and Costa, Garganta, Greco, Mesquita and Maia [50]. These actions were evaluated each second, following the suggestions of Mendes and Malacarne [51]. The two repetitions of the same small-sided games were sequenced and summed, resulting in three 6 min situations for each AMA team and three 6 min situations for each PRO team. The time during which the players analyzed were not playing (i.e., the ball left the pitch or the goalkeeper was controlling the ball) was removed, and all trials were equalized, resulting in an overall analysis of 204 seconds for each type of small-sided games. The interval and ratio scales, i.e. positional data, are maximally objective and due to this property they intrinsically have larger potential for generalization. However, space-time data are not able to provide direct information on which tasks individual players were exploring and in what context, within the game. Because we were interested in the technical-tactical and exploratory behavior in the discrete task-space [52] the nominal scale was the scale of choice. Each 1 s window was defined as a 51-component binary vector representing the full action configuration during the same time interval. A value of 1 was ascribed to the active category and a value of 0 to the inactive one. Both observers analyzed one game (6 minutes) of each team to calculate reliability. The inter-observer agreement yielded a minimum Kappa coefficient of 0.82, providing a satisfactory guarantee of data reliability. One of the observers analyzed the other trials of all teams.

**Table 1:** Observation instrument defined on a coarse-grained scale and based on the technical/tactical actions of players (Adapted from Owen et al. [45] and Costa et al. [53])*.

Role	Technical/tactical action	Definition
**Attacker with ball**	Run to the ball	Player who runs towards the ball with the intention of reaching it
	Wait	Player remains still until the ball is close to where he is, at a point where another action can be performed
	Control	Player receives an intended pass or makes contact in attempting to maintain possession of the ball
	Pass	Player in possession sends the ball to a teammate
	Shoot	Player in possession intentionally sends the ball towards the goal in an attempt to score
	Protect	Putting one’s body between the ball and one or more opponents with the aim of keeping possession
	Drive	Movement of ball carrier towards the goal or changing direction in order to play in other areas of the pitch line
	Feint	Gesture or movement to deceive the opponent
	Dribble	Player in possession, with ball at feet, runs with ball, beats or attempts to beat an opponent
	Intercept	Player makes contact with or stops the ball, enabling him to regain possession and preventing an opponent’s pass from reaching its intended destination
	Deflect	Unintentionally changing the trajectory of the ball after it was kicked by the opponent
	Clear	Intentionally moving the ball away from a zone or situation close to one’s own goal
	Anticipate	Action intending to dispossess an opponent who is in possession of the ball.
**Attackers without ball**	Wait	Remaining in a fixed position or walking, waiting the action of the ball carrier, teammates and opponent
	Support	Player’ movements towards the ball carrier offering a passing option aimed on keeping the ball possession
	Unmark	Movement of players between the last defender and towards the goal line amplifying the effective playing space and offering a long pass option
**Defenders**	Press	Actions to regain the ball or attempt to make the opponent lose the ball
	Delay	Actions to slow down the opponent’s attempt to move forward with the ball
	Dissuade	Take up a position with respect to an opponent without the ball to make it difficult for him to receive or to prevent him from receiving the ball
	Balance	Positioning of off-ball defenders in response to movements of attackers in an attempt to achieve numerical stability or superiority with respect to the opposition
	Withdraw	Move back to a position between the line of the ball and one’s own goal

*The number of players who performed each action (1, 2, 3 or more) was defined each second.

### Data analysis

In order to analyze the influence of the task constraints on the use of the different type and number of actions, a hierarchical principal components analysis [54] under Direct Oblimin rotation, with Delta = 0, aiming data reduction was performed (for the suitability of using principal components analysis with binary variables, see [55]). The number of significant first level principal components was determined by identifying those that accounted for ≥80% of the explained variance. The component correlation matrix of the first-order principal components was then subjected to a further higher-order analysis. The component score matrix was used in order to detect the most salient collective and individual action categories that emerged during play.

To identify a team’s collective exploratory behavior during games, the average dynamic overlap, <*q*_*d*_*(t)*>, was calculated as an average cosine auto-similarity of the overlaps between configurations with increasing time lag (for more details, see Hristovski et al. [7]). This measure captures the average similarity of configurations or game patterns at ever increasing time distances (i.e., time lags) from each other. Hence, it is capable of detecting the rate and breadth of exploratory behavior over different time scales. For example, if a team uses different types of attacking actions (i.e. running to the ball, then control it and then pass it, but in the following attacks the players use other combinations of actions) and defending actions (sometimes press, sometimes delay and others balance, for instance) will have a larger exploratory breadth than a team of players who perform the same actions over time. Had the players remained in the same position or had they repeated the same action throughout the observation time, then the average dynamic overlap would be a constant equal to 1 (i.e., <*q*_*stat*_*>* = 1) for all time lags. On the other hand, if, during the game, the players explored distant configurations of all the possible combinations of actions, then the average overlap (<*q*_*stat*_*)>*) would be zero. This will be detected in a short time scale of some seconds, but also for larger time scales of minutes.

The average dynamic overlap was fitted by the following equation, which is derived for systems with an intricate hierarchical structure [56]:

$$<q_{d}\left(t\right)>=\left(1-q_{stat}\right)t^{\alpha }+q_{stat}$$ (1)

where *q*_*stat*_ is the asymptotic (i.e., stationary) value of the dynamic overlap, *t* is the time lag, and α is the dynamic exponent. *q*_*stat*_ detects the long-term exploratory breadth of the team, and α the rate of exploration. The fit between the theoretical prediction and the experimental data was assessed by the quasi-Newton method of least squares estimation, with the goodness of fit being based on the percentage of explained empirical variance.

In the current study, the sample average <*q*_*stat*_*>* values under different task constraints using a one-way ANOVA were compared across the different scenarios. A statistical significance level of 95% was selected and effect sizes (Cohen’s d) were computed to identify the magnitude of the effects.

## Results

### Principal component analysis

**Fig 2:**
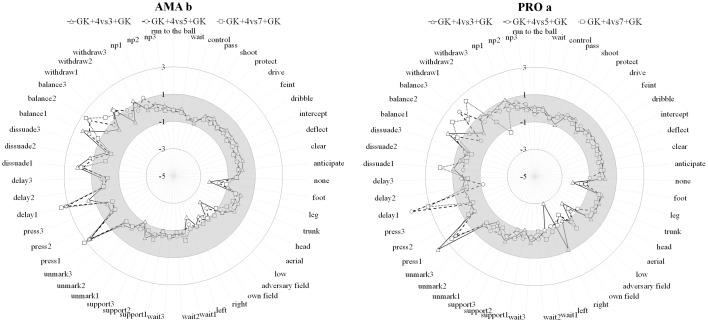
Highest level PCs for AMAb and PROa (fixed teams).

Under the Kaiser-Guttman criterion the hierarchical principal components analysis initially revealed between 9 and 14 primary principal components (PCs) in each file. The second-level PC structure was formed by salient correlated clusters of first-order PCs. A significant dimensional reduction was obtained, resulting in between three and five PCs to analyze. The secondary-level correlated structures systematically produced a sole third-level PCs under the majority of the trials, although a fourth level was present in some cases. By way of an example, the results from this highest level PC for AMAb and PROa (fixed teams) are presented in Fig 2. The highest level PC of the remaining teams was very similar.

These results showed that the essential technical/tactical actions of soccer were the most frequent in all situations and in both AMA and PRO players. When attacking, the most common actions used by the player in possession of the ball were controlling, passing, and driving the ball, whereas waiting or supporting the player with the ball were the actions most used by attacking players without the ball. On the other side, when defending, results showed less variability in the different tactical actions used, being the most common combination: one player pressing, one delaying, one dissuading, and one balancing and/or one withdrawing. Results for the variable team were very similar, with the exception that the number of players without the ball, who performed more technical/tactical actions as the number of players in the team increased.

Due to these similar results for all situations in the highest level PC, which seems to capture the essential characteristics of soccer, it was important to analyze the first set of primary PCs of each trial. For the fixed team, an increase in the number of opponents led to an increase in the time spent defending, this being the case for both PRO and AMA players. A further finding for both levels when defending was a decrease in the number of players dissuading and an increase in the number of players balancing. In 4 vs. 7 games, the most frequent scenario was all the players balancing. In relation to attack, an increase in the number of opponents produced a decrease in passing and driving actions among PRO players, whereas AMA players showed a decrease in passing and controlling actions. For both levels there were fewer players waiting during the game in inferiority. For the variable team, an increase in the number of teammates led to more time being spent in attacking situations. No clear differences appeared with respect to the player with the ball, but an increase in the number of teammates did produce an increase in the number of players waiting and supporting. When defending, there was a clear increase in the number of players involved in balancing. In all the situations, the most frequent combination was one player pressing, one delaying, one dissuading, and/or one or more balancing.

### Exploratory dynamics

**Fig 3:**
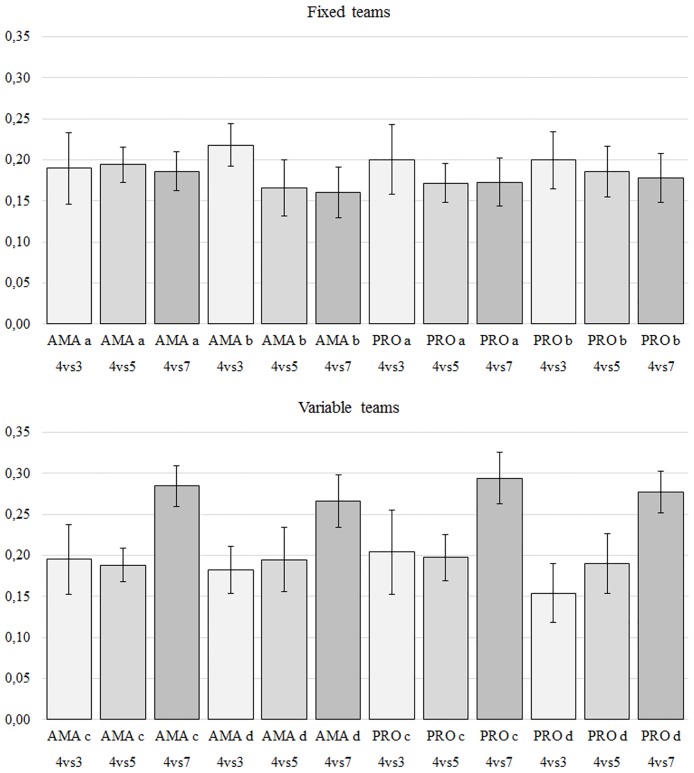
Averages of the stationary overlap order parameter, <*q*_*stat*_>, for each trial.

The goodness of fit between the theoretical curve and the experimental data (n = 102) for the exploratory dynamics (see Eq 1) reached values of R^2^ = .91 ± 0.04. This result confirms that Eq (1) is able to capture the essential hierarchical dynamics during small-sided games. Results from the exploratory dynamics analysis are summarized in Table 2 and Fig 3, where it is presented the averages of the stationary overlap order parameter, <*q*_*stat*_>, for each trial, comparing the values of each team in all conditions. Results show different values of exploration but with clear differences between the different small-sided games. For the fixed teams, AMAa showed a small effect of the number of opponents, just comparing 5 OPP vs 7 OPP. AMAb showed strong effects of the number of opponents when comparing 3 vs 5 OPP and 3OPP vs 7 OPP. PROa and PROb showed moderate effects when comparing 3 OPP vs 5 OPP and 3 OPP vs 5 OPP. In the case of the variable teams, playing with seven teammates clearly produced a lower exploratory breadth compared with the other conditions. All teams showed strong effects of the number of teammates when comparing 5 TM vs 7 TM and 3 TM vs 7 TM.

**Table 2:** Mean values (±SD) for the stationary part of the overlap order parameter (<q_stat_)>) obtained for the three conditions in all teams.

Fixed teams	3 OPP	5 OPP	7 OPP	p (Cohen’s d)	Variable teams	3 TM	5 TM	7 TM	p (Cohen’s d)
**AMAa**	0.19±0.04	0.19±0.03	0.19±0.02	a) -	**AMAc**	0.2±0.04	0.19±0.02	0.28±0.02	d) p < .01 (0.21)
				b) p < .05 (0.36)					e) p < .01 (4.27)
				c) -					f) p < .01 (2.65)
**AMAb**	0.22±0.03	0.17±0.03	0.16±0.03	a) p < .01 (1.73)	**AMAd**	0.18±0.03	0.19±0.04	0.27±0.03	d) -
				b) -					e) p < .01 (2.00)
				c) p < .01 (2.01)					f) p < .01 (2.74)
**PROa**	0.20±0.04	0.17±0.02	0.17±0.03	a) p < .01 (0.87)	**PROc**	0.2±0.05	0.2±0.03	0.29±0.03	d) -
				b) -					e) p < .01 (3.27)
				c) p < .01 (0.76)					f) p < .01 (2.18)
**PROb**	0.20±0.03	0.19±0.03	0.18±0.03	a) p < .05 (0.43)	**PROd**	0.15±0.04	0.19±0.04	0.28±0.03	d) p < .01 (1.01)
				b) -					e) p < .01 (2.8)
				c) p < .05 (0.66)					f) p < .01 (4.02)

Abbreviations: PRO = professional players; AMA = amateurs players; OPP = opponents; TM = teammates. Significant differences are identified as: a) 3 OPP vs. 5 OPP; b) 5 OPP vs. 7 OPP; c) 3 OPP vs. 7 OPP; d) 3 TM vs. 5 TM; e) 5 TM vs. 7 TM; f) 3 TM vs. 7 TM.

## Discussion

This study explored the use of constraints as a way of enhancing players’ exploratory behavior in soccer, and also analyzed the emergence of individual and collective action configurations when varying the number of opponents or teammates in the context of numerically unbalanced teams. The analysis was only focused on observable motor behavior, therefore, disregarding important behavioral factors such as participants’ emotions, thoughts, the influence of the partners’ behavior, or the subjective meaning of their behavior. In addition, the effectiveness of play was not analyzed, mainly due to the controversies found in using any objective measure of overall performance.

The results showed a topological and temporal structure of relatedness between the configurations emerging when playing soccer [7]. In general, certain technical/tactical actions appeared with a high frequency, while others were statistically rare and short-lived reconfigurations that constitute fluctuations following dynamical systems principles [21]. Highly frequent combination of actions loaded predominantly on the first PC, capturing most of the variance. The variation in the number of opponents and teammates had a significant effect on the type of action configurations performed by the players in lower order PCs, although this effect was not appreciated in the highest level PC, which captures the essential characteristics of the game but not the peculiarities of each situation. This result gives a clear picture of the emergent hierarchical organization of soccer, in which the general task constraints of the game give rise to a stable and general structure that is captured by the high similarity of the highest order PCs [57]. However, more immediate and peculiar task constraints, specific to the relative number of players on the pitch and, perhaps, to personal constraints of the players involved in the game, induce a more differentiated structure of decisions-actions at the individual and collective level, and this is captured in lower order PCs.

An increase in the number of opponents produced an increase of the frequency of defensive patterns and, especially, an increase in the number of players controlling and a decrease in the number waiting. An increase in the number of teammates produced an increase in the frequency of attacking patterns. By contrast, an increase in the number of opponents produced a decrease in the use of basic actions such as passing or driving by players in possession of the ball, while an increase in the number of teammates produced more defensive actions focused on protecting the goal, with more players balancing, although it did not produce an increase in the number of players pressing or dissuading. This finding should be taken into account by coaches when designing their training sessions, since it suggests that training in situations of numerical inferiority could improve a team’s defensive skills, not only because of the increased time spent in that situation but also because the players become more active when defending in an attempt to prevent shooting opportunities by the opponents. In light of these results, it is suggested that coaches alternate situations of numerical inferiority and superiority in training sessions by using attacking jokers who play for the team in possession of the ball [58]. This strategy could help coaches to take advantage of the observed effects of inferiority in defense and superiority in attack. In general, compared with full-sided games, small-sided games offer more opportunities for players to practice attacking actions such as passing, shooting, dribbling, heading, or supporting teammates with the ball, as well as other defense-related skills such as intercepting the ball [44]. Moreover, small-sided games provide situations closer to instability [23,41,45], since in full-sided team games the global system is harder to destabilize because some subgroups may be inactive [23].

In terms of the emergence of flexible and fluent behavior, players seem to show more exploratory behavior when playing with a numerical disadvantage. This situation seems to force players to vary their game more, whereas a numerical advantage appears to produce game that is less exploratory and less varied. This was especially evident when playing with seven teammates, since the exploratory breadth was clearly lower in all cases. This result is in agreement with the proposal of Stokes [48,59], who considers constraints not only as barriers or structures but also as a possibility to promote a search in different parts of the problem space. This seems to be a general principle, since it has also been found in individual sports training sessions. For example, in order to enhance exploratory behavior one can induce constraints that suppress more habitual actions lying within the comfort zone [26]. The newly induced constraints effectively release the level of constraints that play a crucial role in generating the habitual behavior. As a consequence, new functional behaviors may emerge [6]. Nevertheless, extreme difficult scenarios probably will produce the contrary effect, and players would not be able to cope with the situation. These results may also be related to that obtained when studying the positioning dynamics of small-sided games [60], as an increase in the number of teammates seemed to promote regularity in spatial organization in AMA teams, although in PRO teams the variation in players’ irregularity ranged from trivial to small. PRO players probably anticipate better the need to optimize collective decision-making. However, by studying individual variables, it has been showed that playing in inferiority produces less varied game patterns, especially in defense [57]. It may be the case that the smaller degree of diversity at the individual tactical behavior level is compensated with an increased diversity at the team level, and vice versa [61]. These possible synergic mutually compensating processes may be a key to understanding the multi-level interdependencies in team sports. That is, each individual performer may reciprocally influence and be influenced by team’s behavior in a multilevel synergic relation [62].

In general, these results suggest that easier game situations can promote more regular and less varied play, while more difficult scenarios force the players to explore the varieties of tactical/technical actions that they can perform. This conclusion is consistent with the results obtained in other contexts, for example, the recent analysis of dance improvisation by Torrents et al. [26]. In that study unusual and more difficult instructional constraints produced a more varied dance and led to greater exploration of movement possibilities. Previous research on tactical creativity has shown that it can be improved by practice, with deliberate play or deliberate practice [63], but to our knowledge, there is no literature related to the type of specific task constraints that can enhance creative behavior. The present study suggests that discomfort may be a good way of enhancing creativity, as has been suggested in other fields such as management, art or creative services [64], but more research is needed to verify that this theory is applicable to sport specific settings. More research is also needed in order to evaluate if there is a curvilinear effect of discomfort on the exploratory behavior in team sports as Hristovski, Davids and Araújo [25] observed in boxing by modifying the risk-of-being-hit constraints.

The effect of the three small-sided games formats seems to be similar for both AMA and PRO players. The effect of varying the number of opponents and teammates on the configurations of actions or exploratory behavior seems to be independent of the players’ level. On the contrary, Silva et al. [39] have found that skill level is determinant in the perception of different possibilities for action when evaluating positioning of the team. This model could be useful for studying the emergence of creative behavior in any sport activity, especially team sports or improvisational situations, and also for exploring the influence of task constraints on the teaching-learning process.
